# Impact of Non-Face-to-Face Teaching with Passive Training on Personal Protective Equipment Use in Health Science Students: A Randomized Controlled Trial

**DOI:** 10.3390/ijerph191912981

**Published:** 2022-10-10

**Authors:** Blanca Rueda-Medina, María Encarnación Aguilar-Ferrándiz, Ana Alejandra Esteban-Burgos, Rosa María Tapia Haro, Antonio Casas-Barragán, Almudena Velando-Soriano, Rocío Gil-Gutiérrez, María Correa-Rodríguez

**Affiliations:** 1 Department of Nursing, Faculty of Health Sciences, University of Granada, 18016 Granada, Spain; 2 Instituto de Investigación Biosanitaria ibs. GRANADA, 18012 Granada, Spain; 3 Department of Physical Therapy, Faculty of Health Sciences, University of Granada, 18016 Granada, Spain; 4 Virgen de las Nieves University Hospital, Andalusian Health Service, 18014 Granada, Spain

**Keywords:** non-face-to-face teaching, passive training, personal protective equipment, health science students, COVID-19, clinical simulation

## Abstract

Background: In the COVID-19 era, there was a call for the transformation of higher education. Universities had to combine non-face-to-face teaching with traditional procedures. This study analyzed the effectiveness and perceived satisfaction in a cohort of health sciences students of non-face-to-face teaching with passive training versus face-to-face teaching with active training in the proper donning and doffing of personal protective equipment (PPE) in a clinical simulation scenario. Methods: A total of 142 participants were randomized into two groups: (a) non-face-to-face teaching with passive training; (b) face-to-face teaching with active training. The proper protocol for donning and doffing PPE was assessed. Students evaluated their skills before and after training and satisfaction with training received. Results: Significant differences were observed for the statements “I felt more confident in donning after receiving this training” (*p* = 0.029) and “I felt more confident in doffing after receiving this training” (*p* = 0.042) in the face-to-face teaching with active training group compared to the non-face-to-face teaching with passive training group, whose number of tasks violated was significantly higher (*p* = 0.020). Satisfaction was significantly higher in the face-to-face and active training group (*p* = 0.004). Conclusions: Face-to-face teaching with active training improves effectiveness and satisfaction more than non-face-to-face teaching with passive training for acquiring skills in donning and doffing PPE properly.

## 1. Introduction

In higher education, research has highlighted the importance of students taking an active role in the learning process rather than being passive recipients of information from teachers [1]. Methods that try to approximate theoretical knowledge with what happens in professional practice has been arise [2]. In particular, the simulation consists of representation of real situations that the students will experience in their future work performance [2,3]. Thus, in simulation scenarios, students must put into practice a series of skills, knowledge and attitudes require for their professional competence [4].

Clinical simulation can be considered a teaching–learning strategy that is becoming increasingly important in the acquisition of skills in health students, by giving students the opportunity to practice their clinical skills and decision making without putting at risk patient’s lives [5,6,7]. Clinical simulation is not based only on the handling of simulation mannequins but on a variety of activities that include role-play or virtual environments [8]. It is valued for its ability to reproduce some of the conditions of clinical practice and enable learners to practice in a safe environment [9].

In the era of the coronavirus disease (COVID-19), there was a call for the transformation of higher education. Universities had to combine non-face-to-face teachings in its degrees in addition to its traditional procedures with the new requirements of non-face-to-face teaching [10]. Currently, although the COVID-19 pandemic is over, non-face-to-face lessons may still need to be conducted due to similar circumstances. Thus, several face-to-face teaching and learning methods should also be available to be implemented non-face-to-face [11]. In this context, it is necessary to examine the effects of implementing non-face-to-face teaching methods in health science students.

On the other hand, the COVID-19 pandemic has highlighted the importance of training health sciences students regarding the use of personal protective equipment (PPE). As future healthcare workers, in high-risk environments, it is obliged to wear personal protective equipment [12], since accurate personal protective equipment use is one of the key practices of infection control [13]. Simulation training in the donning and doffing of personal protective equipment has been proposed as an effective methodology to promote the practice of proper technique [14]. However, the effectiveness of online simulation training in personal protective equipment donning and doffing has not been widely investigated.

In this context, the aim of this study was (i) to assess and compare the effectiveness of non-face-to-face teaching with passive training versus face-to-face teaching with active training in the proper donning and doffing of PPE in a clinical simulation scenario based on a COVID-19 patient, and (ii) to analyze the perceived satisfaction in a cohort of health sciences students. 

## 2. Materials and Methods

### 2.1. Study Design and Participants 

A randomized controlled trial was conducted in 142 health science students (36.6% males and 63.4% females) from 1st (28.2%), 2nd (21.8%) and 3rd (50.0%) academic year taken from the Nursing (32.4%) and Physiotherapy (67.6%) Degrees of the Faculty of Health Sciences of Granada and Melilla (University of Granada, Spain). The mean age of the study cohort was 22.54 ± 6.39 years. Students with no prior exposure to simulation were encouraged to become involved in this study. Participants provided written informed consent after receiving information about the purpose of the study. It was explained to the students that participation was voluntary. Therefore, those who chose not to participate would not be academically disadvantaged. Ethical approval was obtained from the Institutional Review Board of the University of Granada. 

### 2.2. Study Protocol 

Prior to the simulation session, a presentation providing the theoretical background to the scenario was emailed to all students who expressed a desire to participate in the study. Students were randomized using the Oxford Minimization and Randomization computer-supported centralized method OxMar [15] into each of the following groups: (a) non-face-to-face teaching with passive training; and (b) face-to-face teaching with active training. In one group, students received face-to-face teaching regarding PPE, and after that, they had to manage the PPE with active training (use of PPE including cap, isolation gown, gloves, goggles and N95 mask). In this group, the training was carried out by the same teacher with the aim of minimizing intra-group differences. The length of each training was 60 min. In the other group, students received non-face-to-face teaching regarding PPE use, and they received a passive training that include watching some videos and protocols regarding the procedure for donning and doffing PPE. In both teaching and training modalities, the protocols and resources used were based on those developed by the Centers for Disease Control and Prevention (CDC) [16] and the World Health Organization (WHO) 2020 [17]. 

**Figure 1:**
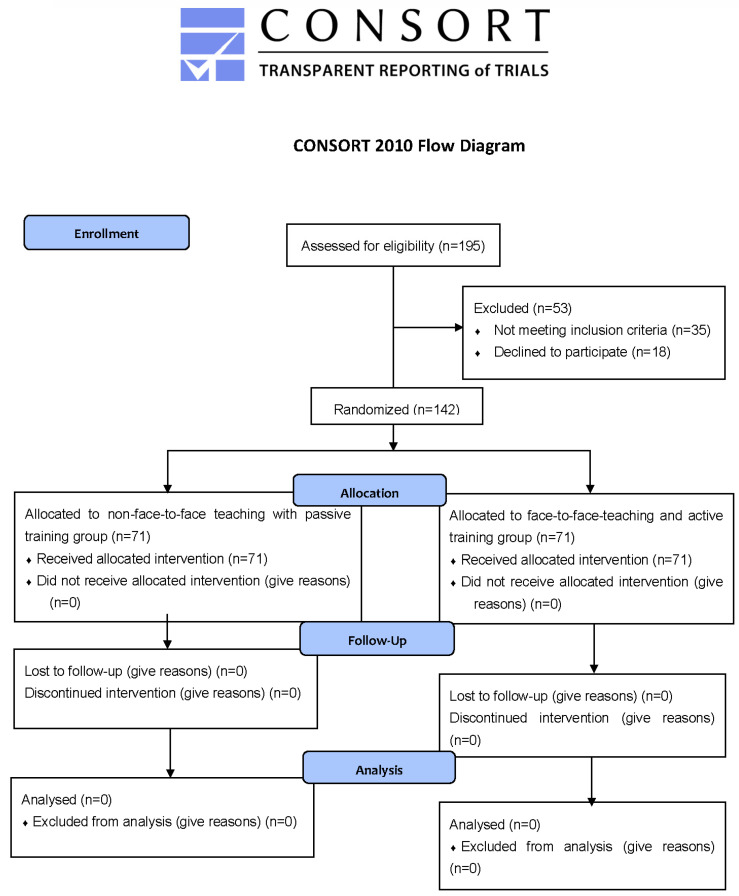
Study protocol.

After the different training modalities, a simulation scenario was designed to evaluate the procedure for donning and doffing PPE. Both groups carried out the same clinical simulation scenario based on the management of a COVID -19 patient. A maximum of 12 participants were admitted in a session. The duration of each simulation with its subsequent debriefing was 90 min (a 15-min simulation and a 75-min debriefing session). The study protocol is shown in Figure 1.

### 2.3. Study Variables 

Students completed a sociodemographic questionnaire including information regarding age, gender, degree, academic year and campus (Granada or Melilla). During the simulation scenario, the proper protocol for donning and doffing PPE was assessed by the instructor using a checklist. Not performing the task or performing it incorrectly was considered an error. Moreover, the total number of donning and doffing tasks violated was calculated. The time to completion for each procedure was also measured.

After the simulation scenario, students completed a self-assessment questionnaire to evaluate their skills in donning and doffing PPE before and after the training received. This questionnaire was previously used by Salway et al. (2020) and consists of a Likert-type scale with values from 1 to 5 (totally disagree and totally agree, respectively). Finally, the perceived satisfaction of the students with the training modalities received was determined using a 10 cm visual analogue scale with scores from 0 (“not satisfied”) to 10 (“very satisfied”). Data were collected between November 2021 and March 2022 without interfering with the participants’ academic degree.

The data were analyzed using SPSS Version 25.0 (IBM Corporation, Armonk, NY, USA). Data were expressed as frequencies and percentages for categorical variables and as mean and standard deviation (SD) for continuous variables. To compare two groups (face-to-face teaching with active training vs. non-face-to-face teaching with passive training), we used Student’s *t*-test for continuous data and χ^2^ for categorical data. All analyses were carried out by a blinded researcher. Probabilities exceeding 95% (alpha *p* values < 0.05) were used as the threshold cut-off for statistical significance.

## 3. Results

### 3.1. Sample Description 

General characteristics of the participants according to the training modalities are shown in Table 1. A total of 142 students (32.4% of the Nursing Degree and 67.6% of the Physiotherapy Degree) participated in the study. Of these, 63.4% of the students were female, and the mean age of the cohort was 22.54 years. No significant differences were found in gender, age, degree, academic year or campus according to the training modalities.

### 3.2. Self-Assessment of the Management of PPE in the Total Study Population According to Training Modalities 

#### 3.2.1. Considering the Response Options as Categorical Variables 

The self-assessment of the management of PPE in the total study population and according to the training modalities is shown in Table 2 and Table 3. Considering the response options as categorical variables, no statistically significant differences were observed between groups (Table 2). Note that before receiving the training, around 40% of students stated that they were familiar with donning and doffing PPE, and 30% of the students considered that they did not have this skill. After receiving the training, almost 100% of the students reported agreeing or strongly agreeing that they were confident donning and doffing the PPE. In addition, more than 95% of the students reported that that the training that they received was useful to manage the clinical simulation scenario.

#### 3.2.2. Considering Means of the Response Options 

The self-assessment of the management of PPE in the total study population and according to the training modalities, considering means of the response options, is presented in Table 3. Significant differences were observed for the statements “I felt more confident in donning after receiving this training” (4.85 (0.39) vs. 4.83 (0.37): *p* = 0.029), and “I felt more confident in doffing after receiving this training” (4.83 (0.37) vs. 4.64 (0.68): *p* = 0.042) in the face-to-face teaching with active training group compared to the non-face-to-face teaching with passive training group. 

### 3.3. Tasks Violated and Time Needed to Accomplish the PPPE Protocol

Table 4 shows the number of donning and doffing tasks violated in the total sample and according to the training received. The task that was completed in a higher percentage was “Put on the isolation gown and tie the straps”, in which an error rate of 9.9% was identified. In contrast, the task that was completed in the lowest percentage was “Put on the mask correctly”, in which an error rate of 22.8% was found. Regarding doffing tasks, it was identified that the 30.3% of students did not complete the task “Take off the gown (untie straps gently, without abrupt movements, from the shoulders down and away from the body”). There were significant differences between the face-to-face teaching with active training group vs. the non-face-to-face teaching with passive training group for the task “Take off the gown” in the doffing procedure (37.2% vs. 62.8% *p* = 0.034). For the rest of the tasks, no statistically significant differences were observed.

Table 5 shows the time needed to accomplish the EPI protocol and the total number of tasks violated between both training modalities. Regarding time to completion, no significant differences were observed between both groups. Interestingly, the total number of tasks violated was significantly higher in the non-face-to-face teaching with passive training groups vs. the face-to-face teaching with active training group (2.23 (1.99) vs. 1.53 (1.78); *p* = 0.029).

### 3.4. Perceived Satisfaction 

With regard to perceived satisfaction, we found a high level of satisfaction in the total population (9.14 (1.32)). However, we identified that the level of satisfaction was significantly higher in the face-to-face teaching with active training group vs. the non-face-to-face teaching with passive training group (9.46 (0.78) versus 8.81 (1.66); *p* = 0.004).

## 4. Discussion

In this study, we aimed to assess and compare the effectiveness of non-face-to-face teaching with passive training versus face-to-face teaching with active training in the proper donning and doffing of PPE in a clinical simulation scenario based on a COVID-19 patient, and to analyze the perceived satisfaction in a cohort of health sciences students. We demonstrated that the total number of tasks violated in the face-to-face teaching with active training group was significantly lower, supporting the greater effectiveness of this methodology to acquire skills in donning and doffing PPE properly. The level of perceived satisfaction was also significantly higher in this group. 

The global trend in higher education to respond to the pandemic era with e-learning options has resulted in the rapid transition from face-to-face and active training to non-face-to-face and passive training [18]. Therefore, it is of special interest to evaluate the effects of implementing non-face-to-face teaching methods in health sciences students. Similar to our findings, previous studies also found that the level of satisfaction was higher in face-to-face vs. non-face-to-face teaching. Results from prior research carried out in the post COVID-19 era on healthcare degrees including nursing, physiotherapy, medicine or dentistry have shown the positive impact on face-to-face training in terms of social performance, knowledge integration, self-motivation, security and satisfaction [19,20,21,22,23]. These studies have been conducted in diverse settings that require both a strong practical component, such as suturing, anatomy or dissection, and a strong social interaction component, such as learning specific terminology for health professionals. This is one of the biggest challenges in non-face-to-face education: not all activities work well in online environments [24], which has to be taken into consideration when designing these activities. Possible factors that may influence greater student satisfaction using active vs. passive methods may be due to the possibility of making decisions and integrating theory with practice, which provides a greater perception of learning, besides promoting social interaction and a beneficial environment during the session and its following debriefing [25]. Much of the success of the success of face-to-face learning approaches is due to instructor-dependent factors, which non-face-to-face interventions cannot replace. Factors as simple as their level of enthusiasm, as well as its external manifestation, has shown to have a direct impact on students’ behavior [26], and they manage to better capture students’ attention [27]. Even details such as the instructor’s voice during the lesson have been demonstrated to influence the learning processing process [28], finding significant differences in retention capacity and time estimates [29]. 

The COVID-19 pandemic has highlighted the relevance of training future health professionals regarding the proper use of PPE in order to stop the infection-spreading process [30,31]. In addition to reducing the risk of infection spread, proper handling of PPEs promotes the physical and mental well-being of healthcare workers. Hoedls et al. [32] showed that in a cohort of nursing professionals, the higher the stress levels, the longer they wore their masks, which must be taken into account both to establish mandatory break periods of the equipment use and to organize adequate training that guarantees an adequate adjustment of the devices with the objective of reducing possible discomfort and guaranteeing security. 

In the context of COVID-19, previous experience in handling PPEs in previous sanitary emergencies such as Ebola, SARS or any other respiratory or infectious conditions can be also supportive. Evidence has shown that active training (face-to-face training) in PPE use decreases non-compliance with donning and doffing guidance more than passive training modalities (lectures, videos, without practice) [33,34]. The use of reality-based education methods, such as clinical simulation, has been shown to help significantly reduce the contamination associated with PPEs’ management [13]. However, while some authors have reported fewer errors using methodologies with active versus passive formations [34], others have not found significant differences in compliance with the protocol [35,36], or even the opposite, finding significant differences in favor of the use of online methodologies (e.g., mobile video) instead of conventional learning strategies in health professionals managing PPEs [31], which may be due to the immediacy (any moment, any time) offered by these modern approaches in a profession marked by constantly changing schedules and routines determined by patient conditions. It is true that both modalities have common features, which can be, among others, the possibility of downloading the content and assignments, as if students were taking notes in a face-to-face lesson, or the possibility of starting a debate or reflection process during lessons remotely thanks to videoconference platforms. However, as some studies have shown, in the case of health science students, the proper students, although accepting online modalities as an alternative in the context of the health emergency caused by the COVID-19 disease [11], strongly believe that face-to-face education is irreplaceable [37]. 

In our study, we observed that the total number of tasks violated was higher in the non-face-to-face teaching with passive training group. Currat et al. [38] also found that adding to a passive training (a gamified e-learning module) an active intervention (face-to-face intervention using Peyton’s four-step approach) in a cohort of 65 paramedic students produced a higher proportion of doffing sequences properly performed compared to the passive intervention group (33.3%, 95% CI 18.0 to 51.8 versus 9.7%, 95% CI 2.0 to 25.8; *p* = 0.03). In addition, the self-assessment of the management of PPE reported by the students in our study has revealed that students felt more confident in donning and doffing after receiving face-to-face teaching with active training. In line with these results, Díaz-Guío et al. [39], conducted a study in 106 medicine students with the objective of comparing the use of an active versus a passive methodology when managing COVID-19 patients and PPE equipment and found that participants who received passive methodology had lower levels of satisfaction. Thus, our results taken together with data from previous reports support that face-to-face teaching with active training before a clinical simulation scenario might ensure the acquisition of skills for the proper PPE use. 

In this study, the task that was completed in a higher percentage by the students was “Put on the isolation gown and tie the straps”, whereas the tasks that were completed in the lowest percentage were “Put on the mask correctly” and “Take off the gown (untie straps gently, without abrupt movements, from the shoulders down and away from the body”)”. Compliance with guidelines and protocols when donning and doffing PPEs has been poor through time [33]. When it comes to the use of respiratory protection (e.g., face masks), compliance is around 50% in many cases [40]. Other studies also have reported a higher number of errors in PPE doffing tasks [34,41,42,43,44]. Based on the results of a Cochrane Database Systematic Review, face-to-face training could reduce noncompliance with doffing guidance protocols more than when only videos or folders are provided to trainees (odds ratio 0.45, 95% CI 0.21 to 0.98) [33]. These findings should be a cause for concern for teachers and students, since the risk of contamination when removing PPE is greater than when putting it on [42]. In fact, the European Center for Disease Prevention and Control (ECDC) guidelines themselves have particular emphasis on proper training for employees on PPE removal [30]. Future training should emphasize that external surfaces when removing PPE are contaminated and focus especially on the acquisition of these skills.

This study has some limitations that should be highlighted. The health science students enrolled in this study were trained to properly use the PPE and therefore, our findings might not be generalized to other clinical scenarios. In addition, since the sample was entirely constituted by health science students, the effects reported in this trial might not be generalizable to other domains and degrees. Although participants did not receive the audiovisual material prior to the intervention, it is possible that some of them accessed and watched the same or similar videos or protocols available online, and this variable was not monitored and controlled for. Finally, we did not assess the difference in the retention rate of skills acquired after training over time between groups, which would be interesting to include in future studies. In contrast, reporting and selection biases were minimized, as this was a prospective, randomized controlled trial in which data collection was not based on participants’ memory or prior information, and aleatory group allocation avoided distorting results based on participants’ personal characteristics. It also should be highlighted that the same experienced teacher completed all the teaching sessions in order to reduce the confounding effect derived from multiple teachers. Moreover, the low risk of detection bias should be noted, as the evaluators of the simulations were blinded to the study groups. 

## 5. Conclusions

In conclusion, our results support that face-to-face teaching with active training is more effective than non-face-to-face teaching with passive training in order to acquire skills in the donning and doffing of PPE properly in health science students. The level of perceived satisfaction by the students was also significantly higher in this group. Educators can use this information to promote face-to-face teaching with active training before clinical simulation scenarios that require the use of PPE, since it appears to improve effectiveness and satisfaction. Further studies in a larger study cohort are required to validate our findings. 

## Figures and Tables

**Table 1:** General characteristics of the participants according to the training modalities.

	Total	Face-to-Face Teaching with Active Training	Non-Face-to-Face Teaching with Passive Training	
	N = 142	N = 72 (50.7)	N = 70 (49.3)	*p* Value
Gender				
Women	90 (63.4)	49 (54.4)	41 (45.6)	0.241
Age (years)	22.54 ± 6.39	23.44 ± 7.49	21.61 ± 4.91	0.088
Degree				
Nursing	46 (32.4)	22 (47.8)	24 (52.2)	0.635
Physiotherapy	96 (67.6)	50 (52.1)	46 (47.9)	
Academic Year				
1st	40 (28.2)	16 (40.0)	24 (60.0)	0.279
2nd	31 (21.8)	17 (54.8)	24 (45.2)	
3rd	71 (50.0)	39 (54.9)	32 (45.1)	
Campus				
Granada	46 (32.4)	22 (47.8)	24 (52.2)	0.635
Melilla	96 (67.6)	50 (52.1)	46 (47.9)	

Data are expressed as frequencies and percentages and as mean and standard deviation (SD).

**Table 2:** Self-assessment of the management of PPE in the total study population and according to the training modalities (response options as categorical variables).

Please Rate the Extent to which You Agree or Disagree with Each of the Following Statements:	Response	Total (N = 142)	Face-to-Face Teaching with Active Training (N = 72)	Non-Face-to-Face Teaching with Passive Training (N = 70)	
		N (%)	N (%)	N (%)	*p* Value
1. I felt confident in donning prior to receiving this training	Strongly Disagree	24 (16.9)	15 (62.5)	9 (37.5)	0.272
	In Disagreement	22 (15.5)	12 (54.5)	10 (45.5)	
	Neutral	31 (21.8)	12 (38.7)	19 (61.3)	
	In Agreement	35 (24.6)	15 (42.9)	20 (57.1)	
	Totally Agree	30 (21.1)	18 (60.0)	12 (40.0)	
2. I felt confident in doffing prior to receiving this training	Strongly Disagree	28 (19.7)	18 (64.3)	10 (35.7)	0.125
	In Disagreement	23 (16.2)	11(47.8)	12 (52.2)	
	Neutral	35 (24.6)	14 (40.0)	21 (60.0)	
	In Agreement	26 (18.3)	10 (38.5)	16 (61.5)	
	Totally Agree	30 (21.1)	19 (63.3)	11 (36.7)	
3. The training that I received explained the steps of donning and doffing adequately for me	Strongly Disagree	1 (0.7)	0 (0.0)	1 (100.0)	0.467
	In Disagreement	2 (1.4)	0 (0.0)	2 (100.0)	
	Neutral	3 (2.1)	2 (66.7)	1 (33.3)	
	In Agreement	27 (19.0)	13 (48.1)	14 (51.9)	
	Totally Agree	109 (76.8)	57 (52.3)	52 (47.7)	
4. I felt more confident in donning after receiving this training	Strongly Disagree	1 (0.7)	0 (0.0)	1 (100.0)	0.177
	In Disagreement	0 (0.0)			
	Neutral	5 (3.5)	1 (20.0)	4 (80.0)	
	In Agreement	23 (16.2)	9 (39.1)	14 (60.9)	
	Totally Agree	113 (79.6)	62 (54.9)	51 (45.1)	
5. I felt more confident in doffing after receiving this training	Strongly Disagree	1 (0.7)	0 (0.0)	1 (100.0)	0.192
	In Disagreement	0 (0.0)			
	Neutral	2 (1.4)	0 (0.0)	2 (100.0)	
	In Agreement	29 (20.4)	12 (41.4)	17 (58.6)	
	Totally Agree	110 (77.5)	60 (54.5)	50 (45.5)	
6. This training capture my attention	Strongly Disagree	1 (0.7)	0 (0.0)	1 (100.0)	0.190
	In Disagreement	2 (1.4)	0 (0.0)	2 (100.0)	
	Neutral	6 (4.2)	5 (83.3)	1 (16.7)	
	In Agreement	27 (19.0)	12 (44.4)	15 (55.6)	
	Totally Agree	106 (74.6)	55 (51.9)	51 (48.1)	

Data are expressed as frequencies and percentage.

**Table 3:** Self-assessment of the management of PPE in the total study population and according to the training modalities (mean of the response option).

Please Rate the Extent to which you Agree or Disagree with Each of the Following Statements:	Total (N = 142)	Face-to-Face Teaching with Active Training (N = 72)	Non-Face-to-Face Teaching with Passive Training (N = 70)	
	Mean (SD)	Mean (SD)	Mean (SD)	*p* Value
1. I felt confident in donning prior to receiving this training	3.18 (1.38)	3.13 (1.49)	3.23 (1.26)	0.656
2. I felt confident in doffing prior to receiving this training	3.05 (1.41)	3.01 (1.54)	3.09 (1.27)	0.762
3. The training that I received explained the steps of donning and doffing adequately for me	4.70 (0.65)	4.76 (0.49)	4.63 (0.78)	0.221
4. I felt more confident in donning after receiving this training	4.74 (0.59)	4.85 (0.39)	4.63 (0.73)	0.029
5. I felt more confident in doffing after receiving this training	4.74 (0.55)	4.83 (0.37)	4.64 (0.68)	0.042
6. This training capture my attention	4.65 (0.69)	4.69 (0.59)	4.61 (0.79)	0.494

Data are expressed as mean and standard deviation (SD).

**Table 4:** Number of donning and doffing tasks violated in the total sample and according to the training received.

	Tasks Violated			
	Total (N = 142)	Face-to-Face Teaching with Active Training (N = 72)	Non-Face-to-Face Teaching with Passive Training (N = 70)	
	N (%)	N (%)	N (%)	*p* Value
Donning Tasks Completed				
1. Hand hygiene	17 (12.0)	12 (70.6)	5 (29.4)	0.080
2. Put on the isolation gown and tie the straps	14 (9.9)	8 (57.1)	6 (42.9)	0.612
3. Put on the mask correctly (mold the metal band to the shape of the nose with your hands and cover up to the bottom of the chin; nose and mouth must be protected)	48 (33.8)	27 (56.3)	21 (43.8)	0.345
4. Put on protective glasses	18 (12.7)	7 (38.9)	11 (61.1)	0.283
5. Put on the gloves (cover the cuffs of the gown, without leaving bare skin on the wrists)	22 (15.5)	11 (50.0)	11 (50.0)	0.943
Doffing Tasks Completed				
1. Remove gloves using the correct technique to avoid hand contamination	34 (23.9)	14 (41.2)	20 (58.8)	0.203
2. Take off the gown (untie straps gently, without abrupt movements, from the shoulders down and away from the body)	43 (30.3)	16 (37.2)	27 (62.8)	0.034
3. Hand hygiene	31 (21.8)	12 (38.7)	19 (61.3)	0.131
4. Remove goggles (pulling the back of the temple and pulling it forward away from the head, without touching the front)	27 (19.0)	10 (37.0)	17 (63.0)	0.114
5. Take off the mask (do not touch the front part)	17 (12.0)	5 (29.4)	12 (70.6)	0.061
6. Hand hygiene	29 (20.4)	14 (48.3)	15 (51.7)	0.769

Data are expressed as frequencies and percentage.

**Table 5:** Time needed to accomplish the protocol in both donning and doffing and the total number of tasks violated between both training modalities.

	Total (N = 142)	Face-to-Face Teaching with Active Training (N = 72)	Non-Face-to-Face Teaching with Passive Training (N = 70)	
	Mean (SD)	Mean (SD)	Mean (SD)	*p* Value
Time for donning (seconds)	120.55 (38.87)	120.90 (40.01)	120.19 (37.95)	0.913
Time for doffing (seconds)	71.46 (24.41)	75.11 (28.53)	67.71 (18.75)	0.070
Total number of tasks violated	1.87 (1.91)	1.53 (1.78)	2.23 (1.99)	0.029

Data are expressed as mean and standard deviation (SD).

## Data Availability

The databases used and/or analyzed during the performance of this study can be provided by the corresponding author through their appropriate request.
